# Infant Hand Preference and the Development of Cognitive Abilities

**DOI:** 10.3389/fpsyg.2016.00410

**Published:** 2016-03-23

**Authors:** George F. Michel, Julie M. Campbell, Emily C. Marcinowski, Eliza L. Nelson, Iryna Babik

**Affiliations:** ^1^Department of Psychology, The University of North Carolina at GreensboroGreensboro, NC, USA; ^2^Department of Physical Therapy, Virginia Commonwealth UniversityRichmond, VA, USA; ^3^Department of Psychology, Florida International UniversityMiami, FL, USA; ^4^Department of Physical Therapy, University of DelawareNewark, NJ, USA

**Keywords:** hand preference, infants, embodied cognition, cognitive development

## Abstract

Hand preference develops in the first two postnatal years with nearly half of infants exhibiting a consistent early preference for acquiring objects. Others exhibit a more variable developmental trajectory but by the end of their second postnatal year, most exhibit a consistent hand preference for role-differentiated bimanual manipulation. According to some forms of embodiment theory, these differences in hand use patterns should influence the way children interact with their environments, which, in turn, should affect the structure and function of brain development. Such early differences in brain development should result in different trajectories of psychological development. We present evidence that children with consistent early hand preferences exhibit advanced patterns of cognitive development as compared to children who develop a hand preference later. Differences in the developmental trajectory of hand preference are predictive of developmental differences in language, object management skills, and tool-use skills. As predicted by Casasanto’s body-specificity hypothesis, infants with different hand preferences proceed along different developmental pathways of cognitive functioning.

## Introduction

For the last four decades, one of us (GFM) has been investigating the factors affecting the development of infant hand-use preferences as a means of understanding how infant sensorimotor experiences contribute to the development of hemispheric differences in language processing (cf., Michel, 1988, 1991). The theoretical foundation of this work was derived, in part, from theories of Piaget (1952) and Bruner (1973), which proposed that infant sensorimotor experiences formed the foundation of symbol formation, language skill, concept formation, and reasoning. Also, developmental psychobiological evidence about how nervous system functioning could be shaped by early experience was used to support the notion that infant hand preferences could shape functional lateralization of the hemispheres (Michel, 1998, 2002). Since most neuropsychological research was, and is, conducted within the framework that hemispheric specialization of function derives from gene controlled differences in the structural organization of the two hemispheres, hand preference and related cognitive functions are often considered to be derivative of hemispheric specialization rather than to be contributors to it. However, in the last decade, some forms of embodiment theory (e.g., Barsalou, 2008; Casasanto, 2009) have reopened theoretical consideration that sensorimotor experiences can contribute to the functional organization of the brain.

Certain forms of embodiment theory (e.g., Barsalou, 2008; Casasanto, 2009, 2011) propose that the development of symbolic cognitive and social knowledge depends on the individual’s sensorimotor engagement with social companions and physical objects during infancy. Infant vocalizations, facial expressions, and body postures (as sensorimotor actions) elicit actions from social companions, which provide the developing infant with information about the rules of social-relational engagement. This is similar to how the manipulation of objects reveals object properties, their relations, and rules of combination. How infants engage with these aspects of their environment sculpts their brain functioning and structure (Boulenger et al., 2009), which, in turn, affects their cognitive and social development. Thus, if there are group differences in such patterns of engagement, then there ought to be group differences in cognitive and social abilities. One way of testing whether such engagements with objects shape cognitive development would be to compare the cognitive development of groups of individuals who engage the world differently during infancy.

We propose that the development of infant hand preferences creates groups of infants who engage the world differently and hence, they should develop differences in cognitive functioning. This thesis is in accordance with Casasanto’s (2011) body-specificity hypothesis and the thesis of this Frontiers’ special issue (contributions of sensorimotor experience to cognitive development). We briefly review some of our studies which demonstrate that consistent infant hand preferences predict developmental advances in language development, tool-use, and objects construction skill. We propose that these advances not only contribute to the individual’s development of language, concept formation, and reasoning, but also the individual’s functional organization of the brain.

## The Hand Preference Phenomenon

Hand preferences in adults are related to differences in hemispheric specialization for language skills (e.g., Corballis, 2009; Häberling et al., 2015), word processing differences between hemispheres (e.g., Willems et al., 2010), and a remarkably wide range of performance differences on tasks of cognitive, social, and emotional functioning (Annett, 2002). Moreover, apparently atypical hand preference development seems to be related to nearly every mental and medical health issue (e.g., Michel et al., 2013b, pp. 207–208). Consequently, some investigators have argued that examination of the cognitive and social abilities of different hand preference groups is the perfect test for evaluating embodiment theory (e.g., Casasanto, 2009). Hand preference represents different patterns of hemispheric specialization and such specialization may be relevant for the manifestation of specific aspects of cognitive, social, and emotional functioning. Therefore, the development of hand preference ought to relate to the typical and atypical development of many psychological functions.

However, before we can understand how hand preferences could contribute to variability in embodied cognitive experiences, we first must understand how hand preferences develop. Hand preference is the product of multifaceted developmental processes that begin before birth and expand during early infancy (Michel et al., 2013b). We have found that hand preferences are developing in a cascading fashion with preferences for earlier developing manual skills (e.g., reaching, grasping/acquisition) concatenating into preferences for later developing skills (e.g., unimanual and bimanual manipulation, artifact construction, and tool-use). We observed that a hand preference for acquiring objects starts manifesting before the age of 6 months, becomes prominent during 6–12 month period, and declines thereafter (Michel, 2002; Ferre et al., 2010; Michel et al., 2014). Also, although unimanual manipulation skills develop by 7–8 months, only by 10–11 months of age do infants exhibit a hand preference for unimanual manipulation and that preference matches the preference for acquisition (Campbell et al., 2015a). By 13–14 months of age, there is a significant increase in hand preferences for role-differentiated bimanual manipulation (RDBM, Babik and Michel, 2016). The hand preference for RDBM seems to stabilize by the age of 18 months (Nelson et al., 2013) with 80% of toddlers maintaining the same preference to 24 months. A consistent hand preference for RDBM likely influences the development of hand preference for tool-use, since RDBM is an object manipulation pattern characteristic of most actions involved in tool construction and use throughout the life-span (Vauclair, 1984). Although a right hand preference predominates in all infant manual skills, the hand preferences appear to be distributed continuously across infants (similarly to adult hand preferences, Annett, 2002).

Although approximately 12% of infants have a consistent left hand preference (Michel et al., 2014), the left hand preference does not appear to be as robust as the right preference. In part, this may be a consequence of a maternal influence on the development of infant hand preferences (Harkins and Michel, 1988; Michel, 1992, 1998; Mundale, 1992, unpublished). Right-handed mothers unintentionally engage the use of their infant’s right hand during object play (Michel, 1998). In contrast, although left-handed mothers use their left hand more when playing with their infants, the difference in their hand use is small compared to the overwhelming use of the right hand by right-handed mothers. Thus, left preference infants of right-handed mothers (the majority of left preferring infants) are likely to be encouraged (by their mother’s actions during socially interactive object play) to use their right hand much more than right preference infants of left-handed mothers (a minority of right preferring infants) are encouraged to use their left hand. Indeed, infants initially manifesting left-hand preference for acquiring objects who had right-hand preferring mothers significantly reduced their left-hand preference by 11 months; whereas infants initially manifesting right-hand preference who had right-hand preferring mothers strengthened their right-hand preference by 11 months (Michel, 1992).

## Assessing Hand Preference Development Using a Trajectory Based Analysis

The expression of infant hand preferences reflects the consequences of an immature but rapidly developing nervous system and expression can vary according to such factors as circadian rhythm, situational arousal, and the development of other neuromotor abilities, such as postural control and locomotion (Corbetta and Thelen, 2002; Babik et al., 2014). Moreover, the identification of a preference appears to be sensitive to various assessment procedures and conditions (Campbell et al., 2015b). Therefore, assessment of infant hand preferences requires longitudinal designs using tasks that are relatively similar, across age, in the manual challenge that they present for the infant. Object acquisition skills can be used to assess the development of hand preferences during the period from 6 to 14 months of age because this manual skill is prevalent in the infant’s repertoire but it is sufficiently challenging to elicit a hand preference across this age period. Moreover, it is incorporated into all other manual skills involving object manipulation (construction of objects and tool use). A hand preference in reaching and object contact predicts the hand preference in object acquisition (Michel and Harkins, 1986) and the hand preference for acquiring objects predicts the hand preference for unimanual object manipulation (Hinojosa et al., 2003; Campbell et al., 2015a) and RDBM (Nelson et al., 2013; Babik and Michel, 2016). Thus, early-developing hand preference for object acquisition is pivotal for the development of hand preference for other more sophisticated manual skills.

We hypothesized that there ought to be consistent developmental trajectories for object acquisition hand preference despite some variation across assessment ages. Nine monthly assessments during the 6–14 month period permitted reliable identification of four latent groups according to their pattern of developmental trajectories using group based trajectory modeling (GBTM, Nagin, 2005; Michel et al., 2013a). GBTM permits identification distinctive patterns in the distribution of a sample’s trajectories to define the infant’s hand preference We found that 32% of 380 infants have consistent right-hand preferences from 6 to 14 months of age, 12% have consistent left preferences, and 26% have a developmental trajectory trending toward a later developing right preference. The remaining 30% of infants had a consistent trajectory of hand use that showed no differences in hand-use across the ages. Hierarchical Linear Modeling (HLM, Raudenbush et al., 2004), confirmed that the infants assigned to these four latent classes exhibited significantly different trajectories in their development of hand preferences. Infants with a right preference have established that preference by 6 months of age and maintain it for the next 9 months (with a slight decrease in right hand use by 13 and 14 months). Infants with a left preference had not established that preference before 8 months of age, but maintain it thereafter. Infants with a trend toward a right preference start at 6 months without a preference but have established a right preference by 14 months. Those infants without a hand preference maintain that throughout the 6–14 month period. Thus, by the beginning of the second year, hand preferences are exhibiting the common character of a right preference for most and a left preference for about 12% of the infants and about 30% with unclear preferences. Of course, the number of groups identified is less important than the recognition that it is only by the collection of such longitudinal data that consistencies across assessment periods can be identified. It is those consistencies of preference that reflect the operation of neural mechanisms that ought to contribute to the development of other mechanisms associated with cognitive functioning.

## Hand Preference and Language

The development of manual skills dynamically shifts the way infants experience their world, and various changes in motor skills have been linked to changes in language ability (e.g., Iverson, 2010). Here, we highlight our longitudinal studies that have used a trajectory-based approach to characterize hand preference and address how hand preference trajectories may be differentially related to language acquisition.

Nelson et al. (2014) hypothesized that a consistent infant hand preference was a marker for advanced object manipulation skill, whereas an inconsistent preference would be an indicator of a lower skill level and perhaps a different pattern of hemispheric specialization. Nelson et al. (2014) described trajectories in the timing and direction of hand preference among children assessed monthly as infants (6–14 months) and then as toddlers (18–24 months): children with consistent right-hand preference as infants who remained right-handed as toddlers, and children without consistent hand preference as infants who became either right-handed or left-handed as toddlers. Consistency versus inconsistency of hand preference from infancy through toddlerhood explained 25% of the variance in language ability at 2 years of age. Also, consistent right-hand preference from infancy was associated with advanced language skills. Gonzalez et al. (2015) extended this work to include language outcome at 3 years in the same sample and found that children with a consistent hand preference trajectory as toddlers had higher expressive language scores. Thus, early, consistent hand preferences may facilitate the development of language (Nelson et al., 2014; Gonzalez et al., 2015). Although more works needs to be done, Michel et al. (2013a) used Arbib’s schema theory (Arbib, 2006) to delineate some of the mechanisms by which the sensorimotor experience associated with a hand preference could contribute to the neural control of expressive language skills.

## Hand Preference and the Manual Control of Objects

It is reasonable to assume that infants with a hand preference for engaging with objects would develop greater manual skill and proficiency with the preferred hand and that preference would affect the development of their manual control of objects. Object construction requires manually merging multiple objects into a single, unifying structure, such as stacking blocks into a tower or assembling a puzzle (Marcinowski, 2015). Object construction has recently been related to a variety of cognitive skills at later ages, including mathematical ability (Wolfgang et al., 2003; Nath and Szucs, 2014), language (Marcinowski and Campbell, 2015) and visuospatial skill (Caldera et al., 1999; Levine et al., 2011; Verdine et al., 2014).

Marcinowski (2015) found that infants with a consistent hand preference develop stacking more quickly during 10–14 months period than infants without a hand preference. Consistent left- and right-preferring infants manifested greater stacking skill at 14 months, than infants without a consistent hand preference. Moreover, infants with a trending right preference did not differ in the development of their stacking skill from infants without a preference. Since the trending right group did not exhibit a hand preference for acquisition during the 6–9 months period before stacking began to be assessed, they likely had not developed the manual proficiency needed to stack objects (Chen et al., 2010). Thus, the consistency of a hand preference changes the relation between a hand preference and the cognitive skill of object construction.

Also, Kotwica et al. (2008) reported that infants with consistent hand preferences across four assessment periods (at 7, 9, 11, and 13 months of age) are more effective with the object management skill of object storage than infants without a consistent preference during that period. When infants are given multiple toys (one at a time), they must develop the ability to manipulate and manage these objects so that the latter are available for future interaction. Infants with consistent hand preference demonstrated a greater skill for object storage, such as placing objects in reachable locations and intermanual transfer, than infants without a hand preference (Kotwica et al., 2008). By storing objects more effectively, infants with a hand preference can explore properties of objects, understand relations between objects, and “plan” actions more effectively than infants without a preference (cf., Bruner, 1973). Indeed, Bruner (1973) considered object storage skills to be important for the development of symbolic representation (and hence language development), since an unused, but stored object must be mentally represented by the infant for later retrieval.

Tool use is another important cognitive skill that often involves imitation of complex actions, planning, decision-making, and the ability to account for spatial and temporal characteristics of objects, their properties, and the situation. Many have argued that tool use requires advanced symbolic thinking and representational means-end analysis (Bates et al., 1980), advanced causal understanding (Carpenter et al., 1998; Buttelmann et al., 2008), and an achievement of spatial reasoning that permits coordinating multiple mobile frames of reference (Lockman, 2000). Fraz et al. (2014) tested the development of the tool use skill longitudinally from 10 to14 months in 60 infants with right, left, or no hand preference for acquiring objects. They found that infants with consistent right or left hand preference out-performed those without a hand preference in the number of successfully completed tool-using actions at the ages of 10, 11, and 12 months. However, after 12 months differences between the hand preference groups ceased to be statistically significant. Fraz et al. (2014) concluded that early-development of consistent hand-use preferences for acquiring objects facilitates the onset of the cognitive skill of tool use. Thus, we have shown how longitudinal assessments of the consistency of hand preferences relate to the development of the manipulation of objects that are considered to contribute to the development of symbolic cognitive abilities.

## Conclusion

We have demonstrated that early-established hand preferences (revealed by their consistency across longitudinal assessments) for object acquisition and manipulation of objects significantly predict developmental advancement of such important elements of cognitive development as expressive language, object construction, object management skills, and tool use. Thus, it is important that longitudinal consistency in infant hand preferences be taken into account while exploring patterns of neurobehavioral functioning and cognitive development. Our results are consistent with the predictions of Casasanto’s (2009, 2011) body-specificity hypothesis. Having a more practiced, preferred hand could assist infants in scaffolding their manual proficiency and hence their comprehension of the properties of objects. Such comprehension, in turn, could contribute to the development of other cognitive abilities as revealed in object construction, tool-using, and language development. What remains to be demonstrated is how these differences in hand preference development have influenced the functional organization of the infant’s brain.

## Author Contributions

JC, EM, EN, IB, and GM conceptualized and wrote the paper.

## Conflict of Interest Statement

The authors declare that the research was conducted in the absence of any commercial or financial relationships that could be construed as a potential conflict of interest.
